# Contribution of PPi-Hydrolyzing Function of Vacuolar H^+^-Pyrophosphatase in Vegetative Growth of *Arabidopsis*: Evidenced by Expression of Uncoupling Mutated Enzymes

**DOI:** 10.3389/fpls.2016.00415

**Published:** 2016-03-31

**Authors:** Mariko Asaoka, Shoji Segami, Ali Ferjani, Masayoshi Maeshima

**Affiliations:** ^1^Laboratory of Cell Dynamics, Graduate School of Bioagricultural Sciences, Nagoya UniversityNagoya, Japan; ^2^Department of Biology, Tokyo Gakugei UniversityTokyo, Japan

**Keywords:** *Arabidopsis thaliana*, H^+^-pyrophosphatase, pyrophosphate, proton pump, vacuole, plant growth

## Abstract

The vacuolar-type H^+^-pyrophosphatase (H^+^-PPase) catalyzes a coupled reaction of pyrophosphate (PPi) hydrolysis and active proton translocation across the tonoplast. Overexpression of H^+^-PPase improves growth in various plant species, and loss-of-function mutants (*fugu5*s) of H^+^-PPase in *Arabidopsis thaliana* have post-germinative developmental defects. Here, to further clarify the physiological significance of this important enzyme, we newly generated three varieties of H^+^-PPase overexpressing lines with different levels of activity that we analyzed together with the loss-of-function mutant *fugu5-3*. The H^+^-PPase overexpressors exhibited enhanced activity of H^+^-PPase during vegetative growth, but no change in the activity of vacuolar H^+^-ATPase. Overexpressors with high enzymatic activity grew more vigorously with fresh weight increased by more than 24 and 44%, compared to the wild type and *fugu5-3*, respectively. Consistently, the overexpressors had larger rosette leaves and nearly 30% more cells in leaves than the wild type. When uncoupling mutated variants of H^+^-PPase, that could hydrolyze PPi but could not translocate protons, were introduced into the *fugu5-3* mutant background, shoot growth defects recovered to the same levels as when a normal H^+^-PPase was introduced. Taken together, our findings clearly demonstrate that additional expression of H^+^-PPase improves plant growth by increasing cell number, predominantly as a consequence of the PPi-hydrolyzing activity of the enzyme.

## Introduction

H^+^-translocating pyrophosphatases catalyze a coupled reaction of inorganic PPi hydrolysis and active proton transport across membranes (Maeshima, 2000; Martinoia et al., 2007). The presence of two types of H^+^-PPases, type I and type II, has been reported for various organisms (Drozdowicz and Rea, 2001). Every isoform of H^+^-PPases and its related bacterial Na^+^-PPases (Malinen et al., 2007) consist of a single polypeptide with approximately 700–800 amino acid residues (Maeshima, 2001). Type I H^+^-PPases require relatively high concentrations of K^+^ to express their maximal activity, but the type II enzymes do not (Drozdowicz and Rea, 2001). *Arabidopsis thaliana* has three genes encoding H^+^-PPases: a single gene for the type I enzyme, *AtVHP1;1* (Vacuolar H^+^-translocating Pyrophosphatase; AGI locus ID, At1g15690; also called *AVP1*), and two genes for the type II enzyme, *AtVHP2;1* (At1g78920) and *AtVHP2;2* (At1g16780). The type I enzyme AtVHP1 is exclusively localized on the tonoplast (Segami et al., 2010). Both AtVHP2;1 and AtVHP2;2 enzymes are localized in the Golgi apparatus and the *trans*-Golgi network and their total amount is less than 0.3% of that of AtVHP1 (Segami et al., 2010). Therefore, most studies have been focused on the type I enzyme.

Several research groups reported that the overexpression of H^+^-PPase improved plant growth, total mass yield, nutrient-use efficiency, and tolerance to salt and drought stresses in various plant species (Gaxiola et al., 2001; Li et al., 2005; Guo et al., 2006; Brini et al., 2007; Yang et al., 2007; Liu et al., 2011; Vercruyssen et al., 2011; Arif et al., 2013; Pizzio et al., 2015). Based on the above reports, genes for H^+^-PPases have been considered to be useful for growth improvement in plants because of their simple structure. In other words, gene manipulation is easy compared with multi-subunit enzymes. However, the copy number of the transgene and the actual expression levels in the transgenic plants varied significantly within the above studies. As a result, improvement of plant growth and/or stress tolerance was not consistent and differed among the plant species and the transgenic lines.

In most reports, the advantage of overexpressing H^+^-PPases was attributed to the increased capacity of solute uptake into vacuoles and enhanced osmoregulatory capacity across the tonoplast. However, it has remained ambiguous why the loss-of-function mutants of H^+^-PPase showed relatively normal growth (Ferjani et al., 2011; Segami et al., 2014). The vacuolar pH of loss-of-function mutants of vacuolar-type H^+^-ATPase (V-ATPase) was 0.5 point higher than that of wild-type (WT, hereafter) plants and the corresponding mutant plants showed severe growth reduction (Krebs et al., 2010), whereas the pH in loss-of-function mutants of H^+^-PPase such as the *fugu5* mutants increased by only 0.2 point (Ferjani et al., 2011). In both reports, WT had a vacuolar pH of around 5.8 (Krebs et al., 2010; Ferjani et al., 2011).

Although, the proton pumping activity is assumed to be the major physiological role of H^+^-PPase, recent studies using genuine loss-of-function mutants elegantly demonstrated that the opposite is likely true, unraveling novel aspects of the H^+^-PPase (Ferjani et al., 2011). Indeed, functional analyses of the *fugu5* mutant series had unambiguously shown that PPi hydrolysis is essential for active gluconeogenesis to sustain post-germinative growth of *Arabidopsis* seedlings (Ferjani et al., 2011). Thus, leading to the conclusion that the hydrolysis of cytosolic PPi, rather than vacuolar acidification, is the major function of H^+^-PPase during the early stages of plant development, at least under standard growth conditions.

In plant cells, PPi is a by-product released from nearly 200 distinct biosynthetic processes, such as macromolecule biosyntheses of DNA, RNA, proteins and cellulose, and β-oxidation of fatty acids (Maeshima, 2000; Heinonen, 2001; Ferjani et al., 2014). The requisite of PPi hydrolysis, and its link to the phenotype of loss-of-function mutants of H^+^-PPase were clearly demonstrated in post-germinative early developmental stages. So far, analyses of H^+^-PPase overexpressors for the estimation of plant yield or stress tolerance have been done on later growth stages. Provided that, more attention should be paid to the substantial differences in the physiological role(s) of H^+^-PPase between young and mature tissues, since this enzyme is more highly expressed in proliferating young tissues (Shiratake et al., 1997; Nakanishi and Maeshima, 1998; Segami et al., 2014). Henceforth, physiological roles of H^+^-PPase should also be carefully examined during the mid- and late-stages of vegetative growth using loss-of-function mutants of the enzyme and its overexpressors as counterparts.

In this study, to examine the activities of H^+^-PPase and V-ATPase on the growth of plants during early and late growth stages, we prepared three transgenic lines overexpressing a single insertion of *2 × 35S*Ω*_pro_::AtVHP1* at different expression levels to examine the relationship between the enzyme activity and plant growth. We also used the previously established *fugu5-3* as the loss-of-function mutant of H^+^-PPase (Ferjani et al., 2011), and uncoupling mutated variants of H^+^-PPase enzymes that were able to hydrolyze PPi but unable to translocate protons, which were prepared and expressed in the *fugu5-3* background. In a previous study the soluble type PPase, which was localized in the cytosol, was introduced into the mutant under the control of *AtVHP1* promoter (Ferjani et al., 2011). Here, we made constructs for Ile-549-Ala and Leu-753-Ala of H^+^-PPase as typical uncoupling variants and introduced them the *fugu5-3* background to evaluate the PPi hydrolysis activity on the cytoplasmic side of tonoplast. Finally, we discuss the significance of PPi hydrolysis and proton pumping functions during early and late vegetative growth stages.

## Materials and Methods

### Plant Materials and Growth Conditions

Seeds of *Arabidopsis thaliana* (strain, Columbia-0) provided by the RIKEN Bioresource Center (Tsukuba, Japan) were sterilized, placed in the dark at 4°C for 2 days, and then germinated on sterile gel plates containing half-strength Murashige-Skoog (MS) salt, 2.5 mM MES-KOH, pH 5.7, 1% (w/v) sucrose, and 0.5% Gellan gum (MS plates; Wako Pure Chemical, Osaka, Japan) at 22°C under long-day conditions (light/dark regime of 16 h/8 h, cool-white lamps, 90 μmol/m^2^ s). Plants were grown on MS plates without sucrose, when indicated. To obtain mature plants, we transferred WT and transgenic plants to pots set with rockwool blocks (rockwool pots; Nittobo, Tokyo, Japan) and kept in a growth room at 22°C under the above long-day condition. The pots were irrigated twice a week with MGRL medium (Naito et al., 1994; Segami et al., 2014).

### Accession Numbers

Sequence data are provided in the GenBank/EMBL/DDBJ libraries or the *Arabidopsis* Genome Initiative database (http://www.ddbj.nig.ac.jp/index-e.html) under the following accession numbers: *Arabidopsis* gene of H^+^-PPase (*VHP1*; also called *AVP1*/*FUGU5*; At1g15690.1), a gene for H^+^-PPase of *Vigna radiata* (mung bean; AB009077.1), and a T-DNA insertion mutant of H^+^-PPase, *vhp1-1* (Kazusa DNA Research Institute; reference no., KG8420). The *vhp1-1* line was selected and provided by Dr. Yoichi Nakanishi (Nagoya University).

### Generation of Transgenic Plants

For construction of H^+^-PPase overexpressors, the coding region of *VHP1* was amplified from an *Arabidopsis* cDNA library using KOD plus Neo (Toyobo, Osaka, Japan) using a pair of oligonucleotides (**Supplementary Table S1**). The amplified PCR product was cloned into pENTR-D-TOPO (Invitrogen, Carlsbad, CA, USA) and its sequence was confirmed. pENTR-VHP1 was subcloned into the pGWB502Ω binary vector, which possesses 2× CaMV35SΩ-promoter (Nakagawa et al., 2007), by using Gateway LR Clonase II Enzyme Mix (Invitrogen).

For construction of complementation lines with uncoupling mutated variants of H^+^-PPase (Asaoka et al., 2014), we used a *pENTR-VHP1pro::VHP1* plasmid, which had its own *VHP1* promoter prepared previously (Segami et al., 2014). The QuikChange site-directed mutagenesis kit (Stratagene, La Jolla, CA, USA) was used to introduce the nucleotide substitution into the *VHP1* gene to make mutated H^+^-PPases, namely Ile-549-Ala and Leu-753-Ala. Sequences of primers used for site-directed mutagenesis are listed in **Supplementary Table S1**. The DNA sequences of the constructs [*pENTR-VHP1pro::VHP1*] for both native and mutated enzymes (Ile-549-Ala and Leu-753-Ala) were confirmed and were subcloned into the pGWB501 binary vector (Nakagawa et al., 2007).

Each binary vector was transformed into *Agrobacterium tumefaciens* strain C58C1. Several types of *pENTR-VHP1pro::VHP1* constructs were transformed into WT and *fugu5-3* plants by the floral dip method (Clough and Bent, 1998). Transformed plants were selected by the segregation ratio of seedlings on selective media, confirmed to be homozygous for a single T-DNA inserted before use for subsequent in depth analyses.

### Crude Membrane Preparation

Shoots were homogenized at 4°C in a mortar with three volumes of homogenizing medium containing 0.25 M sorbitol, 50 mM Tris-acetate, pH 7.5, 1 mM EGTA-Tris, 1% polyvinylpyrrolidone, 2 mM DTT, and protease inhibitor cocktail (1× Complete, EDTA-free; Roche Applied Science, Mannheim, Germany). The homogenate was centrifuged at 10,000 × *g* for 10 min at 4°C. The supernatant was centrifuged at 100,000 × *g* for 10 min at 4°C. The obtained pellet was suspended in 20 mM Tris-acetate, pH 7.5, 0.25 M sorbitol, 1 mM EGTA-Tris, 1 mM MgCl_2_, 2 mM DTT, and the protease inhibitor cocktail, and used as the crude membrane fraction. Protein content was determined using a protein assay kit (Bio-Rad, Hercules, CA, USA).

### SDS-PAGE and Immunoblotting

Proteins were separated by SDS-PAGE and transferred to an Immobilon-P membrane (Millipore, Billerica, MA, USA). The primary antibodies used were prepared previously: antibodies to H^+^-PPase (antigen peptide sequence, DLVGKIERNIPEDDRN; Segami et al., 2010), V-ATPase subunit A (TKAREVLQREDDLNEI; Kobae et al., 2006), and TIP1s (GVQEEVTHPSALRA; Ferjani et al., 2013), which are available from Agresera (Vännäs, Sweden) and Cosmo Bio (Tokyo, Japan). Chemiluminescent reagent ECL (GE Healthcare, Piscataway, NJ, USA) was used for detection of antigens. Chemiluminescence was detected with a Light-Capture II imaging device equipped with a cooled CCD camera (Atto, Tokyo, Japan). The reproducibility of the results was confirmed in two independent experiments.

### Enzymatic Activity Assays

Pyrophosphate hydrolysis was measured as described previously (Maeshima and Yoshida, 1989). The assay medium for PPi hydrolysis activity contained 1 mM MgCl_2_, 1 mM Na_4_PPi, 1 mM Na_2_MoO_4_, 50 mM KCl, 30 mM Tris-MES, pH 7.2, 0.02% Triton X-100 and crude membranes (800 ng) from plants or vacuolar membranes (300 ng) from *Arabidopsis* suspension cultured cells (refer to methods in supplemental data). The ATP hydrolysis activity of V-ATPase was measured as described previously (Matsuura-Endo et al., 1990; Schumacher et al., 1999) in a reaction medium containing 0.1 mM Na_2_MoO_4_, 50 mM KCl, 0.03% Triton X-100, 3 mM ATP, 3 mM MgSO_4_, 30 mM Tris-MES, pH 7.2, and 2 μg of crude membranes. The activity was determined in the presence or absence of 100 mM KNO_3_ and the nitrate-sensitive activity was defined as V-ATPase activity. Data from at least three independent measurements were averaged.

### Microscopic Observation and Phenotypic Analyses

Leaves were fixed with formalin/acetic acid/alcohol cocktail and cleared with chloral hydrate solution to measure leaf areas and cell numbers (Ferjani et al., 2011). Whole leaves were observed and photographed using a stereoscopic microscope (M165FC; Leica Microsystems, Heidelberg, Germany) equipped with a CCD camera (DFC300FX; Leica Microsystems). Leaf palisade tissue cells were observed and photographed using a stereoscopic microscope (DM-2500; Leica Microsystems) equipped with Nomarski differential interference contrast and a CCD camera (DFC310FX; Leica Microsystems). The cell size was determined as the average cell area of palisade cells, which were observed from the paradermal view as described previously (Ferjani et al., 2011).

### Quantification of Sucrose

The amount of sucrose in seedlings was measured with an ion chromatography system (DX-500; Thermo Fisher Scientific, Waltham, MA, USA) using a CarboPAC^TM^ PA1 column as described previously (Ferjani et al., 2011). Four hundred frozen seedlings were homogenized in chilled 80% ethanol, vortexed for 10 min, treated at 80°C for 30 min, and then centrifuged at 20,000 × *g* for 5 min. The precipitate was resuspended in chilled 80% ethanol and centrifuged. The first and second supernatants were combined and dried by a centrifugal evaporator. An aliquot of water was added into the tube, and water-soluble compounds were extracted. The extract was filtered with a Sep-Pak Light C18 filter (Waters, Milford, MA, USA) and then with a Dismic-13cp (Advantech, Tokyo, Japan). Data from at least three independent measurements were averaged.

## Results

### Preparation and Characterization of Overexpressors with Different Levels of H^+^-PPase Activity

**FIGURE 1:**
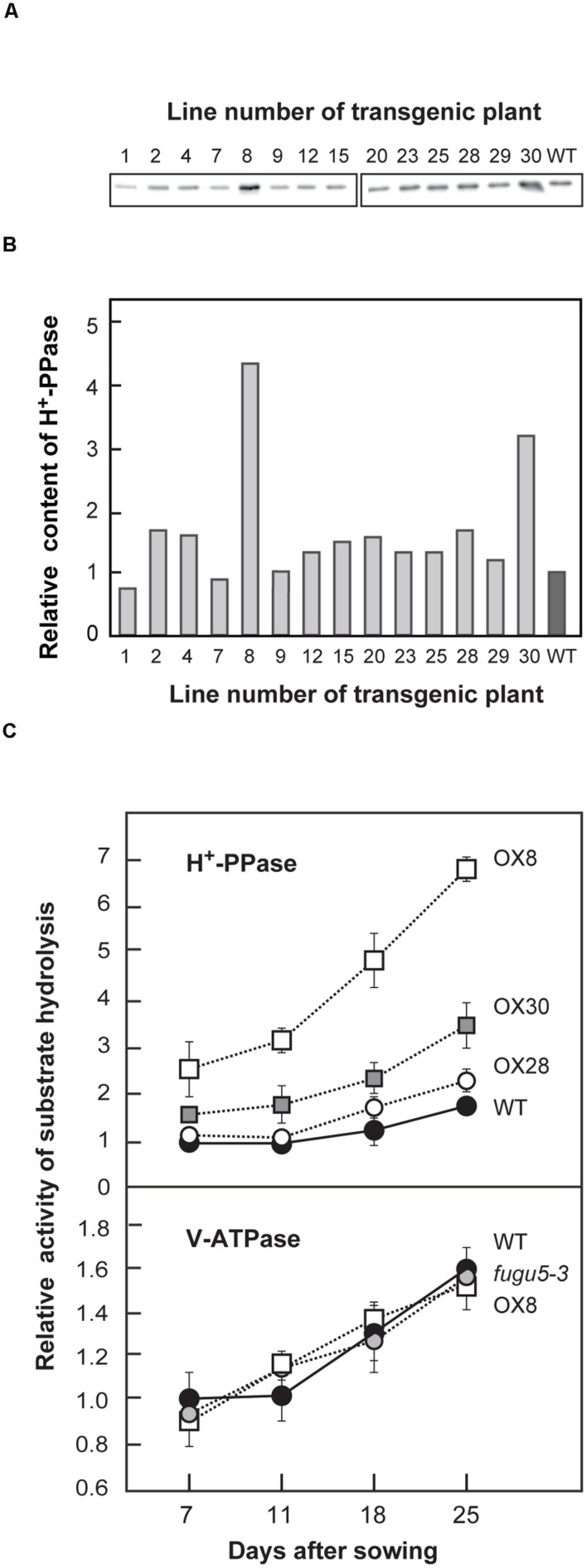
**Expression level and enzyme activities of H^+^-PPase and V-ATPase in WT and mutant lines.** H^+^-PPase overexpressors were grown on MS plates for 15 days **(A,B)** or WT, *fugu5-3* and three representative H^+^-PPase overexpressors (OX8, OX28, and OX30) were grown on MS plates with 1% sucrose for 11 days, transplanted to rockwool pots, and then grown for another 14 days **(C)**. **(A)** Crude membrane fractions were prepared and aliquots (0.5 μg) were subjected to immunoblot analysis with specific antibodies against H^+^-PPase. Representative blots from two independent experiments are shown. **(B)** The intensities of immunostained bands shown in **(A)** are expressed as relative values to those of WT. **(C)** Crude membrane fractions were prepared from plants (0.4–1.0 g of fresh weight) at indicated growth stages and subjected to assays of PPi hydrolysis activity by H^+^-PPase and ATP hydrolysis activity by V-ATPase. Bars indicate SD from three independent experiments. The activities are shown as relative values on the basis of membrane protein to that of WT at 7 DAS. DAS, days after sowing.

We introduced a *2 × 35S*Ω*_pro_::VHP1* construct into WT to evaluate the effect of H^+^-PPase overexpression on plant growth. Then, we selected 14 independent lines in the T3 generation that were both homozygous for a single T-DNA insertion and grew normally under standard growth conditions. The amount of H^+^-PPase protein in shoots was quantified by immunoblotting (**Figures 1A,B**). Among the 14 transgenic lines, OX8, OX28 and OX30, showed relatively high expression levels compared to the WT. OX8, OX30, and OX28 were selected as representative lines with high-, mid-, and low-expression levels, respectively.

Next, we examined the growth of *vhp1-1, fugu5-1*, *fugu5-2*, and *fugu5-3* loss-of-function mutants of H^+^-PPase together with the above three H^+^-PPase overexpressors. The *fugu5-1* (Ala-709 to Thr), and *fugu5-2* (Glu-272 to Lys) mutants consist of a single amino acid substitution. The *fugu5-3* line has both an amino acid substitution (Ala-533 to Thr) and a deletion of five residues (554th to 558th), and *vhp1-1* is a T-DNA insertion line (Ferjani et al., 2011). Despite the various molecular lesions, all four mutants are null alleles and show almost the same gross morphologies, growth and cellular phenotypes (Ferjani et al., 2011). Here, *fugu5-3* was selected as the representative mutant line because it does not accumulate H^+^-PPase protein in the tonoplast, as previously reported (Ferjani et al., 2011). This characteristic should be essential to discriminate native and exogenously introduced (either native or mutated) H^+^-PPases, their expression levels and physiological contributions.

Before in depth examination of the physiological properties of H^+^-PPase overexpressors, PPi hydrolyzing activity was initially measured on crude membranes from WT, *fugu5-3*, OX8, OX28, and OX30 at four distinct growth stages, namely 7, 11, 18, and 25 DAS. In *Arabidopsis*, the period from germination to 11 DAS is generally regarded as an early growth stage. In the present study, 18 and 25 DAS were set as mid- and late-stages of vegetative growth, respectively.

Our measurements revealed that PPi hydrolysis activity of WT increased by ∼70% in the late versus early stage (**Figure 1C**). The OX8 line, which had the highest H^+^-PPase protein content, already had 2.5- and 4-fold higher activities than that of WT at 7 and 25 DAS, respectively (**Figures 1A–C**). The H^+^-PPase activity in the other two lines, OX28 and OX30, increased moderately but was lower than that of OX8 at 25 DAS. It is noteworthy that the H^+^-PPase protein levels in OX8, OX28, and OX30 correlated with the levels of PPi hydrolysis activity (**Supplementary Figure S1A**).

In addition to H^+^-PPase, plant vacuolar membranes usually contain another proton pump, V-ATPase (Maeshima, 2001). In order to check the effect of overexpression of one pump on the other, the relative V-ATPase activity and the protein amounts of its subunit A were determined (**Figure 1C**, **Supplementary Figure S1B**). Both V-ATPase activities and protein levels remained constant in WT, *fugu5-3* and all overexpressors at all growth stages examined (**Figure 1C**, **Supplementary Figure S1B**). On the other hand, the protein levels of the tonoplast aquaporins, TIPs, were basically similar in all genotypes up to 18 DAS, although slightly perturbed at 25 DAS (**Supplemental Figure S1C**). Together, our results clearly show that neither the overexpression nor the loss-of-function of H^+^-PPase affected the levels of the other major tonoplast proteins.

### Contribution of H^+^-PPase in Early Stage of Growth

**FIGURE 2:**
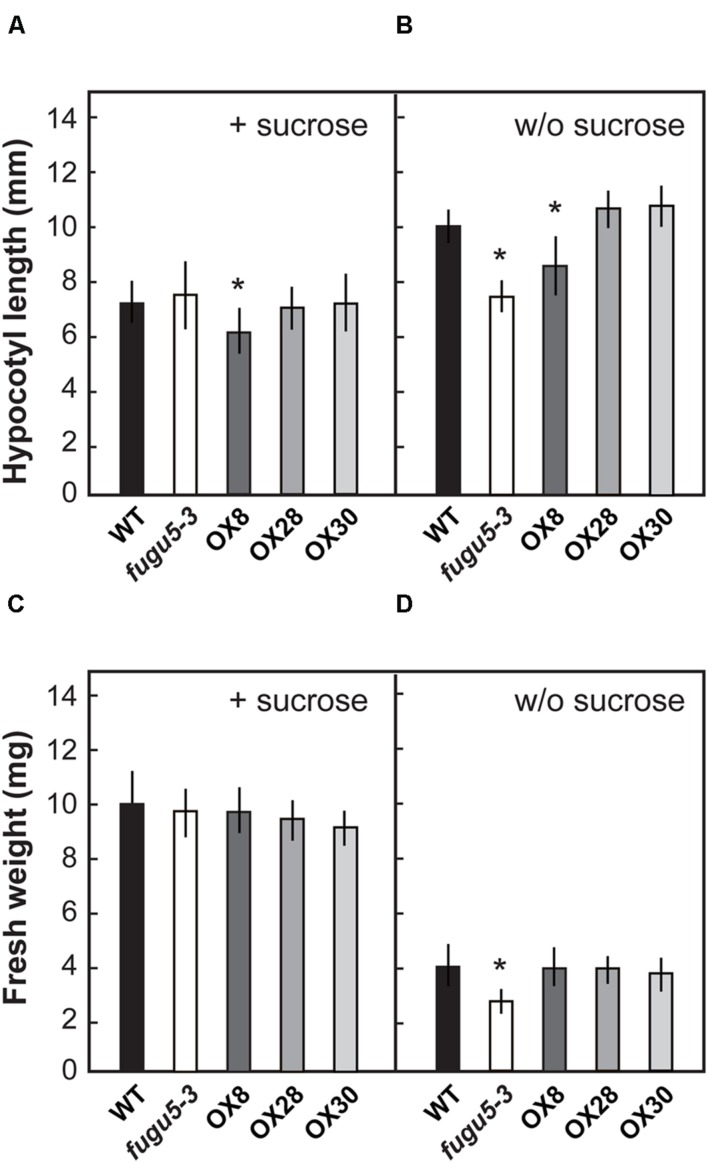
**Growth phenotype of H^+^-PPase overexpressors at an early growth stage.** Seeds of WT, *fugu5-3*, OX8, OX28, and OX30 were germinated on MS plates with 1% sucrose **(A,C)** or without sucrose **(B,D)**. **(A,B)** Length of hypocotyls of etiolated seedlings grown for 3 days (*n* = 50). **(C,D)** Fresh weight of shoots grown for 11 days under long-day conditions (*n* = 40). Error bars show SD. Asterisks indicate significant difference at *P* < 0.05 compared to the WT by two-sided Student’s *t*-test.

The effects of loss of H^+^-PPase function at early growth stages have been investigated and reported in *fugu5* (Ferjani et al., 2011). However, H^+^-PPase overexpressors were not subjected to such analysis. Here, in order to characterize the physiological properties of H^+^-PPase overexpressors, we focused on the early growth stage of plant seedlings. In addition, exogenous supply of a carbon source, such as sucrose, has been reported to rescue the morphological, cellular and growth defects of *fugu5* cotyledons (Ferjani et al., 2011). Therefore, the length of hypocotyls was determined on 3-days-old etiolated seedlings grown with or without 1% sucrose in the growth medium (**Figures 2A,B**). The length of *fugu5-3* hypocotyls was 70% that of the WT, but recovered on media containing 1% sucrose, which is in agreement with a previous report (Ferjani et al., 2011). Also, while the hypocotyls of OX8 were shorter than that of WT by 16 and 18% in the presence or absence of sucrose, respectively, the other overexpressors did not differ significantly from the WT under either growth condition.

Measurements on 11-days-old plantlets revealed that the *fugu5-3* shoot fresh weight was reduced by 34% in the absence of sucrose but recovered to the WT levels when sucrose was supplied (**Figures 2C,D**). Interestingly, fresh weights of OX8, OX28, and OX30 were comparable to the WT irrespective of growth media composition. These results indicated that different degrees of overexpression of H^+^-PPase did not ameliorate seedling growth at an early developmental stage.

### Overexpression of H^+^-PPase Has No Significant Effect on Size and Number of Cotyledon Cells

**FIGURE 3:**
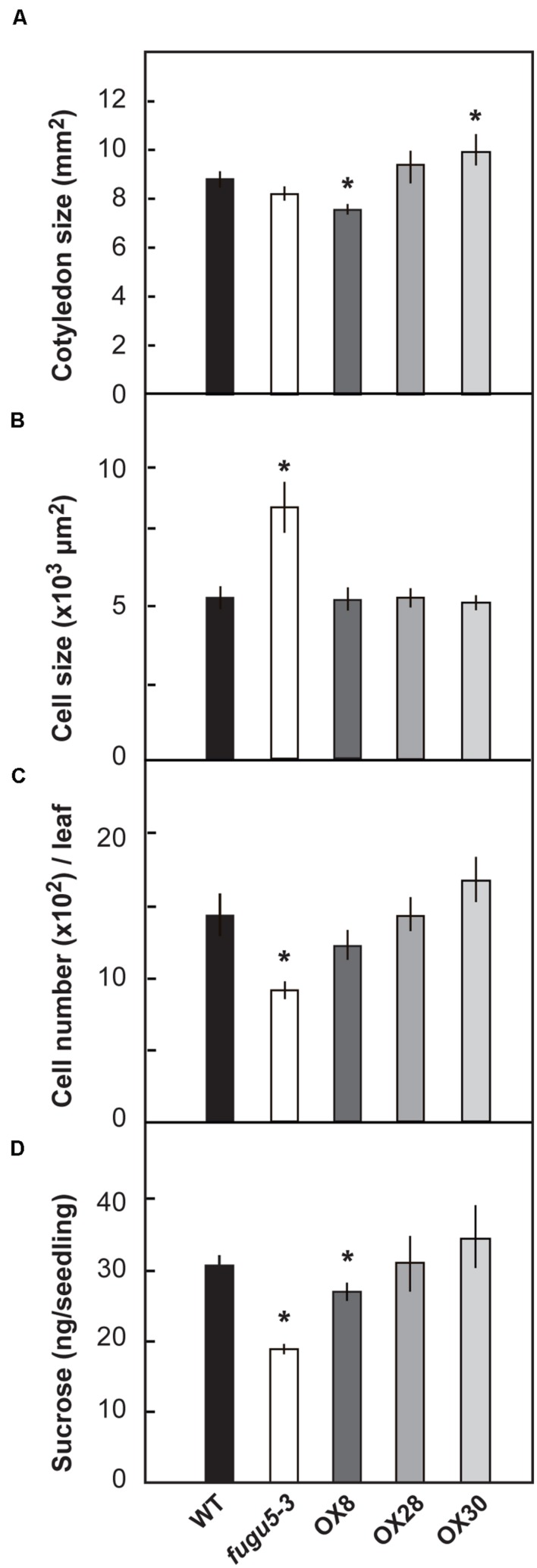
**Effect of H^+^-PPase overexpression on growth of cotyledons.** WT, *fugu5-3*, OX8, OX28, and OX30 plants were grown on the rockwool pots for 25 days. Then the surface area of cotyledons **(A)**, cell size **(B)**, and cell number **(C)** were determined. Error bars show ±SD (*n* = 5). Asterisk indicates significant difference at *P* < 0.05 compared to WT. **(D)** Sucrose amount in 3-days-old etiolated seedlings grown on MS plates without sucrose. Error bars show SD (*n* = 5). 400 etiolated seedlings per each genotype and per each single measurement. Asterisks indicate significant difference at *P* < 0.05 compared to WT (Student’s *t*-test).

“Compensation” is the unusual enhancement of post-mitotic cell expansion triggered by decreased cell number in leaf primordia (Tsukaya, 2002; Hisanaga et al., 2015). The *fugu5* mutants were first identified as compensation exhibiting mutants (Ferjani et al., 2007). Provided that, the number and size of palisade tissue cells of mature cotyledons (at 25 DAS) were determined in WT, *fugu5-3* and the three overexpressors (**Figure 3**). As expected, *fugu5-3* cotyledons contained fewer but larger cells compared to the WT (**Figures 3B,C**), although the surface area of cotyledons did not differ significantly (**Figure 3A**). This is a typical compensation phenotype and agrees with our previous findings (Ferjani et al., 2011). No significant difference was observed in cellular phenotype of overexpressors (**Figures 3B,C**). Among the three overexpressors, OX30 tended to have larger cotyledons due to their increased cell number, although there was no statistical significance compared to the WT (**Figure 3C**).

The loss of H^+^-PPase activity has been also reported to partially compromise gluconeogenesis, which is the conversion of seed storage lipids into sucrose (Ferjani et al., 2011). In the present experiments, 3-days-old etiolated seedlings of *fugu5-3* and OX8 grown without sucrose had a sucrose content 62 and 81% that of WT, respectively (**Figure 3D**). The quantification of triacylglycerols (TAG, hereafter) in dry seeds and their mobilization after seed imbibition revealed no differences among these lines (**Supplementary Figure S2**). Thus, the lower contents of sucrose observed in *fugu5-3* and OX8 are not merely due to the initially low TAG contents in dry seeds, but rather reflect an axial role of H^+^-PPase in gluconeogenesis.

### Stimulation of Growth in Overexpressors at Late Stage of Vegetative Growth

**FIGURE 4:**
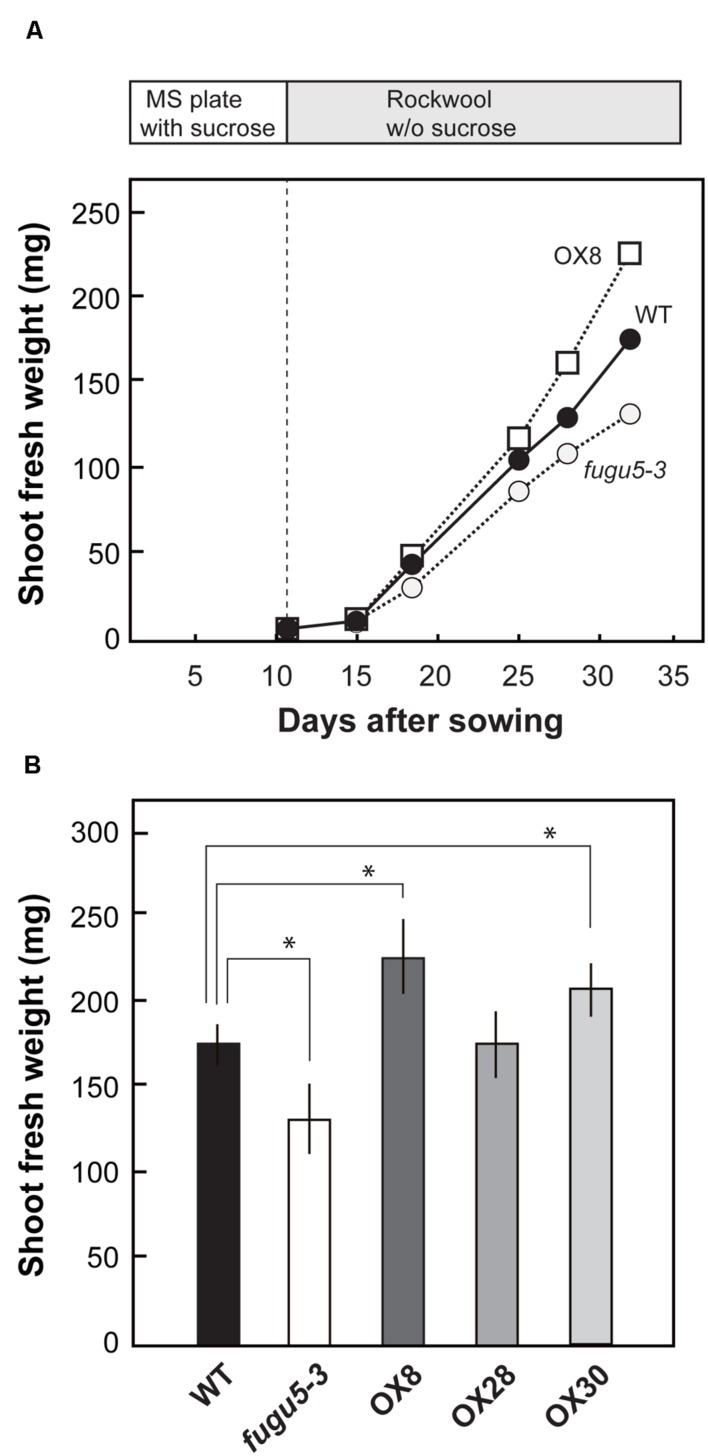
**Growth phenotype of overexpressors in late growth stage.** Seedlings were grown on MS plates with 1% sucrose for 11 days, transplanted to rockwool pots, and then grown for another 21 days. The pots were irrigated twice a week with MGRL medium containing no sucrose. **(A)** Shoot fresh weight of WT, *fugu5-3*, and OX8 grown for indicated period; *n* ≥ 20. **(B)** Shoot fresh weight of 32-days-old plants (*n* = 25). Error bars show SD. Asterisk indicates significant difference at *P* < 0.05 compared with WT (Student’s *t*-test).

We grew seedlings on MS plates containing 1% sucrose for 11 days and then transplanted them into rockwool pots, with no sucrose supplied, and carefully monitored their growth. On 15 DAS, shoot fresh weight was similar in WT, *fugu5-3* and H^+^-PPase overexpressors (**Figure 4A**). An obvious difference in plant growth was observed at 18 DAS. In fact, on 32 DAS, the fresh weight of *fugu5-3* was 26% lower than that of WT (**Figure 4B**). Similar growth defect was also observed for the other mutant alleles of H^+^-PPase, *fugu5-1*, *fugu5-2*, and *vhp1-1* (**Supplementary Figure S3A**). The lower fresh weight in *fugu5-3* recovered upon continuous sucrose supply in the growth media (**Supplementary Figure S3B**). While on the contrary, the weight of OX8 was 30% higher than that of WT at 32 DAS (**Figure 4B**), OX30 also showed significant increase with 18% enhancement and OX28 showed no increment in growth. These results strongly suggest that the loss-of-function and overexpression of H^+^-PPase affect negatively and positively, respectively, plant growth at the late stage of vegetative growth.

**FIGURE 5:**
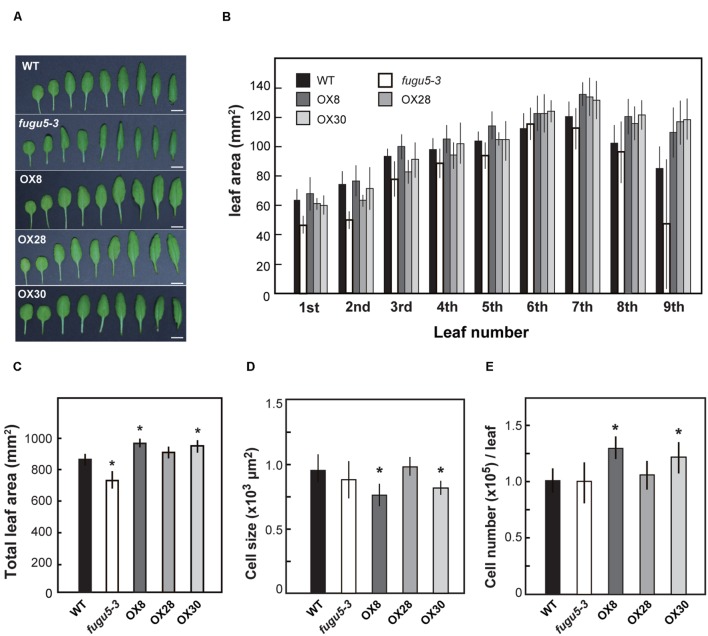
**Growth of leaves in late stage.** WT, *fugu5-3* and overexpressors were grown on MS plates with 1% sucrose for 11 days and then on rockwool pots for another 14 days. Leaves were collected from 25-days-old plants and their leaf surface areas were measured. **(A)** Rosette leaves were taken from WT and mutant lines and were ordered from youngest to oldest ones from the left to the right. Bar = 5 mm. **(B)** Surface area of 1st to 9th rosette leaves. **(C)** Sum area of all rosette leaves of each genotype. **(D)** Cell size of 7th rosette leaves. **(E)** Cell number of 7th rosette leaves. Error bars show SD; *n* = 8. Asterisk indicates significant difference at *P* < 0.05 compared with WT (Student’s *t*-test).

On the other hand, to determine the effects of loss-of-function and overexpression of H^+^-PPase on leaf development, we examined the leaves from the 1st to the 9th positions at 25 DAS (**Figures 5A,B**). While the total surface area of all nine leaves of OX8 and OX30 lines was ∼12% larger, the area of *fugu5-3* was about 15% smaller than that of WT (**Figure 5C**). This is consistent with the differences we found in shoot fresh weight (**Figure 4**). Also, the leaf surface in the three overexpressors showed a particularly significant increase in the rosette leaves from 7th to 9th positions. Yet, this could also reflect the fact that these leaves are not yet fully expanded at 25 DAS.

Next, we determined the number and size of palisade mesophyll cells in the 7th leaves (**Figures 5D,E**). Although *fugu5-3* and OX28 were almost indistinguishable from WT, OX8 and OX30 contained more cells that tended to be slightly smaller than those in WT. Such increase in cell number in OX8 and OX30 might explain their enlarged leaf surface areas (**Figures 5D,E**). Finally, we examined whether the increased levels of H^+^-PPase enhanced the tolerance to salt stress or growth of Pi deficient condition. However, our results revealed no significant improvement were observed on neither salt stress at 50 and 100 mM NaCl (**Supplementary Figure S4**) nor Pi lacking conditions (**Supplementary Figure S5**) in the overexpressors and transgenic lines expressing uncoupling H^+^-PPases, which is described in the next section.

### Involvement of PPi Hydrolysis Activity of H^+^-PPase in Plant Growth Evidenced by Expression of Mutated Uncoupling H^+^-PPases

We examined the PPi hydrolysis function of H^+^-PPase by a unique strategy using newly constructed transgenic lines expressing uncoupling type mutated H^+^-PPase in *fugu5-3* background. These lines were compared with *fugu5-3* and other complementation lines expressing native H^+^-PPase.

Recently, we generated mutant varieties of H^+^-PPases of mung bean (*Vigna radiata*), in which Ile-545 was substituted with Ala (Ile-545-Ala) and Leu-749 with Ala (Leu-749-Ala), respectively. These two mutated H^+^-PPases exhibited normal activity of PPi hydrolysis, but totally lost their H^+^ pumping activity (Asaoka et al., 2014). These mutated H^+^-PPases were named “uncoupling” H^+^-PPases. Amino acid sequences of H^+^-PPase are quite conserved between mung bean and *Arabidopsis.* Residues Ile-545 and Leu-749 in mung bean H^+^-PPase correspond to Ile-549 and Leu-753 in *Arabidopsis* enzyme. We constructed two mutated H^+^-PPases of *Arabidopsis* (Ile-549-Ala and Leu-753-Ala), respectively.

**FIGURE 6:**
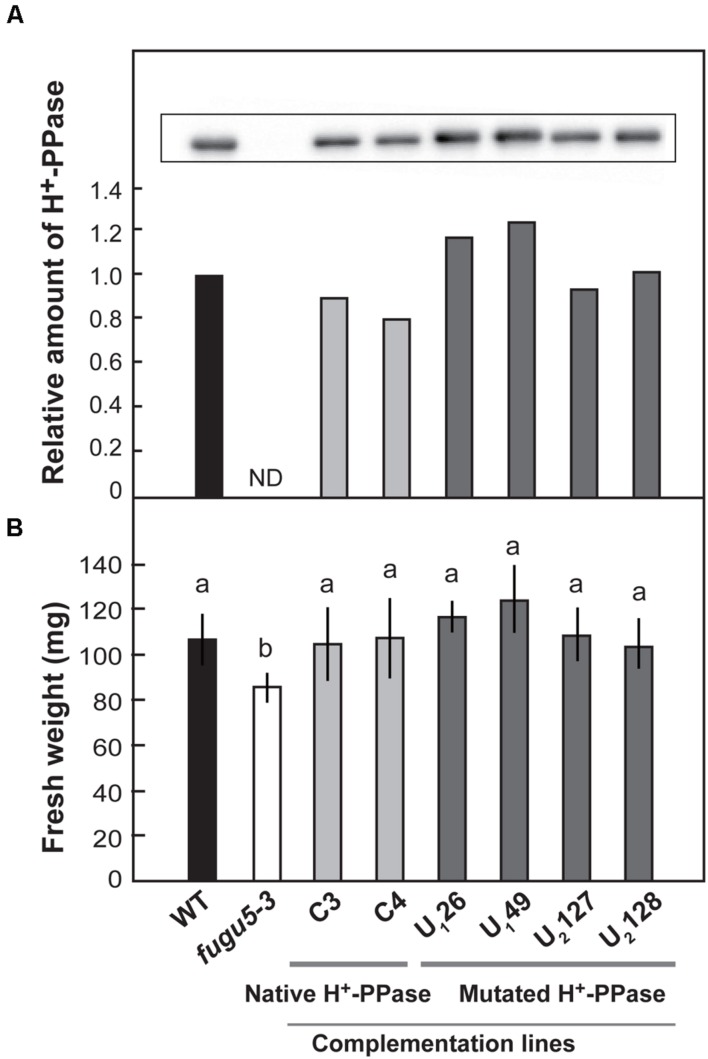
**Effect of introduction of uncoupling mutated H^+^-PPase on growth of *fugu5-3*.** Native (C3 and C4) and uncoupling mutated H^+^-PPases with a single amino acid substitution, Ile-549-Ala (U_1_26 and U_1_49) and Leu-753-Ala (U_2_127 and U_2_128), were transformed into f*ugu5-3.* Single DNA inserted T3 homozygous lines were examined. **(A)** Crude membrane fractions were prepared from seedlings grown on the MS plates with 1% sucrose for 18 days. H^+^-PPase content in the crude membranes was determined by immunoblot analysis. **(B)** Plants were grown on MS plates with 1% sucrose for 11 days and then on rockwool pots for further 14 days. Fresh weight of 25-days-old shoots was measured. Error bars show SD; *n* = 20. Same letters (a) indicate no significant difference at *P* < 0.05 (Steel-Dwass test).

We introduced native and mutated H^+^-PPase into *fugu5-3* background and obtained six stable lines expressing native H^+^-PPase (C3 and C4), Ile-549-Ala (U_1_26 and U_1_49), and Leu-753-Ala (U_2_127 and U_2_128). Immunoblotting analyses confirmed that the native and mutated H^+^-PPase proteins accumulated in each line (**Figure 6A**). Shoot fresh weight was significantly lower in *fugu5-3* than in WT when grown in plates without sucrose. The fresh weight of two transgenic lines (C3 and C4) expressing the native H^+^-PPase was totally recovered (**Figure 6B**). Amazingly, four complementation lines U_1_26, U_1_49, U_2_127 and U_2_128, were also perfectly recovered (**Figure 6B**).

To test the activity of mutated enzymes we used suspension-cultured cells because they provide a relatively high activity of H^+^-pump compared with the membranes prepared from plant tissues (Segami et al., 2014). As shown in **Supplementary Figure S6**, the mutated enzymes in transgenic lines U_1_26 (Ile-549-Ala) and U_2_128 (Leu-753-Ala) were confirmed to be uncoupling H^+^-PPase in the living cells.

Taken together, the expression of uncoupling H^+^-PPases complemented plant growth in *fugu5-3* to the same level as when the native H^+^-PPase was expressed. These results indicated that the H^+^-pump activity of H^+^-PPase is not necessary for the recovery of growth defects of *fugu5-3* mutant.

## Discussion

### Overexpression of H^+^-PPase Is Not Effective in Early Growth Stage

Numerous studies have been conducted on the physiological roles of H^+^-PPase (Maeshima, 2000; Gaxiola et al., 2007). The physiological effects of overexpression of H^+^-PPase on growth and tolerance to multiple kinds of stresses have been reported for *Arabidopsis* (Gaxiola et al., 2001; Li et al., 2005; Yang et al., 2007, 2014; Liu et al., 2011; Pizzio et al., 2015), *Suaeda salsa* L. (Guo et al., 2006), *Nicotiana tabacum* (Li et al., 2014), and *Hordeum vulgare* (Schilling et al., 2014). For example, transgenic barley expressing *Arabidopsis* H^+^-PPase showed increased shoot biomass and grain yield compared with WT when grown under salt stress (Schilling et al., 2014). The extent of enhancement of growth and stress tolerance depended on the plant species and the expression level of the gene encoding H^+^-PPase.

H^+^-PPase is abundant in young growing tissues including proliferating cells (Maeshima, 2000; Segami et al., 2014). The H^+^-PPase protein accounts for about 10% of tonoplast protein of the hypocotyl in mung bean (*Vigna radiata*; Nakanishi and Maeshima, 1998; Maeshima, 2001). H^+^-PPase is also a major tonoplast proton pump in young cells in mung bean (Nakanishi and Maeshima, 1998) and young pear fruits (Shiratake et al., 1997), and the physiological contribution of another proton pump V-ATPase increases as the amount of H^+^-PPase decreases with tissue maturation. Therefore, the physiological contribution(s) of H^+^-PPase in growth might quantitatively and qualitatively change during development of tissues and organs.

At least in metabolically highly active young plant cells, PPi hydrolysis by H^+^-PPase is essential for normal growth of plants because PPi is produced from the synthetic processes of biomolecules, such as DNA, RNA, proteins, cellulose, and sucrose. Hydrolysis of PPi in the cytosol, where most of the above processes occur, is a key function of H^+^-PPase, in addition to its proton pumping function. However, it remained ambiguous whether the H^+^-PPase actually acts as a physiological scavenger of PPi. Recently, the accumulation of PPi at excess levels in the *fugu5* mutant has been demonstrated to partially compromise sucrose synthesis *de novo* (Ferjani et al., 2011). PPi is released by multiple steps during sucrose synthesis from triacylglycerol in germinating seeds. The present study confirmed such a role of H^+^-PPase. In fact, the lack of H^+^-PPase resulted in defect of sucrose supply from cotyledons to hypocotyls and consequently the hypocotyl growth was markedly restrained (**Figure 2**).

We also investigated the effect of H^+^-PPase overexpression on vegetative growth at early stages. OX8 and OX30 had increased levels of H^+^-PPase protein and activity, but the overall growth of etiolated seedlings (**Figure 2**) or cotyledons was not enhanced (**Figure 3**). Here, we should note that the extremely high activity of H^+^-PPase in OX8 (fourfold versus WT) somehow exerted a moderate negative effect on the hypocotyl elongation of etiolated seedlings (**Figure 2**) and on their sucrose contents (**Figure 3**). At present we cannot conclude which function of H^+^-PPase, PPi hydrolysis or proton pump, causes such a negative effects. As a possibility, excessive expression of H^+^-PPase may have direct or indirect negative effect on membrane traffic and/or homeostasis in OX8. This issue needs to be addressed in future studies by comparing the growth of OX8 and transgenic lines overexpressing a soluble-type PPase. In short, the present results suggest that the H^+^-PPase levels in WT are sufficient to sustain optimal growth in early growth stages, and that additional expression of H^+^-PPase does not necessarily have positive effects on growth at this particular stage.

### Overexpression Promotes Growth in Later Stages through Increasing Cell Number

In contrast to early growth stages, overexpression of H^+^-PPase stimulated growth as evidenced from increased shoot fresh weight at 32 DAS (**Figure 4**). Also, the growth rate of the OX-lines positively correlated with H^+^-PPase expression levels (**Figures 4** and **5**). In this study, we selected single-copy insertion lines of *2 × 35S*Ω*_pro_::VHP1* to eliminate side effects of multi-copy insertion of the gene and investigated which process was enhanced by additional expression of H^+^-PPase.

Quantification of cell size and cell number in young rosette leaves indicated that the increase in cell number complements the decrease in cell size and contributes to enlargement of total leaf size (**Figures 4** and **5**). Comparative analysis of transgenic *Arabidopsis* lines that produce enlarged leaves revealed that the increase in leaf area depended on the leaf position and growth conditions and that an increase in cell number was responsible for the leaf size increase (Gonzalez et al., 2010). Previously, growth stimulation of the H^+^-PPase overexpressors was explained by a stimulation of the translocation of auxin in the tissues (Li et al., 2005). The distribution of auxin in *fugu5* mutants was not different from that in WT (Ferjani et al., 2011). The growth of the H^+^-PPase overexpressor was also investigated in relation to the action of *CYTOKININ OXIDASE/DEHYDROGENASE3* (*CKX3*), which catalyzes the degradation of active cytokinin and acts in the process of cell proliferation in meristems (Vercruyssen et al., 2011). It has been concluded that the growth stimulation of the overexpressor does not depend on CKX3. Therefore, the relationship between phytohormones and the cell number increment observed in the rosette leaves of overexpressors (**Figure 5**) should be further examined with respect to the levels of PPi in the cytosol.

### PPi Hydrolysis Activity Rescues Growth and Cellular Defects in *fugu5-3*

The significant decrease in fresh weight and leaf surface area indicated a defect in growth of *fugu5-3* (**Figures 4** and **5**). In the present study, the uncoupling mutated H^+^-PPases were introduced into the *fugu5-3* mutant to examine which function of the enzyme, PPi hydrolysis or proton pumping, is physiologically significant in the plant. The growth of *fugu5-3* totally recovered following the introduction of uncoupling mutated H^+^-PPases (in U_1_26, U_1_49, U_2_127, and U_2_128 transgenic lines) to the same level as *fugu5-3* transformed with the native H^+^-PPase (in C3 and C4 transgenic lines; **Figure 6**). These findings clearly indicated that PPi hydrolysis by H^+^-PPase is important for normal plant growth.

Introduction of yeast soluble inorganic pyrophosphatase IPP1 has been shown to rescue all the developmental and growth defects of *fugu5* (Ferjani et al., 2011). The present study clearly demonstrated that the mutated H^+^-PPase lacking proton pumping function could also similarly rescue the *fugu5* growth defects. In contrast to soluble PPases, the uncoupling mutated H^+^-PPases are localized in the tonoplast and the molecular activities of PPi hydrolysis of the native and mutated enzymes are estimated to be markedly lower (Nakanishi et al., 2003; Hirono et al., 2007; Asaoka et al., 2014). Thus, together with previous reports the present study clearly revealed that the PPi hydrolysis function of H^+^-PPase is preferentially important for normal growth of shoots during vegetative growth. These results support the hypothesis that PPi should be kept at relatively low levels to prevent the inhibition of biosynthesis of macromolecules such as DNA, RNA, proteins, and cellulose in growing cells.

In several organisms, the lack of pyrophosphatase activity is lethal as previously reported for *Saccharomyces cerevisiae* (Lundin et al., 1991). In *Arabidopsis*, in addition to H^+^-PPase, there are six soluble-type PPase isoforms, five of which are predicted to be cytosolic (Schulze et al., 2004; George et al., 2010; Öztürk et al., 2014, 2015). Therefore, the existence of soluble PPases may complement the loss-of-function of H^+^-PPase and protect the mutant plants from serious damage caused by excess PPi. The physiological relationship or contribution of H^+^-PPase and soluble PPases should be investigated to understand how PPi levels are regulated in plant cells. Recent studies revealed a particularly high expression of H^+^-PPase in several tissues or cell types, and its physiological significance has been discussed (Mohammed et al., 2012; Segami et al., 2014; Pizzio et al., 2015). Collective analyses of H^+^-PPase, soluble PPases and V-ATPase are needed to understand the overall physiological role of H^+^-PPase.

## Conclusion

Two major findings were obtained. First, the overexpression of H^+^-PPase improved growth at the late stage of vegetative growth, although not as much as reported previously. Second, the PPi hydrolysis, rather than the proton pumping function, is the physiologically significant function of H^+^-PPase that secures optimal plant growth at least in *Arabidopsis*.

## Author Contributions

MA co-coordinated the project, contributed to preparation of transgenic lines and phenotyping, analyzed data, and drafted the manuscript; SS conducted the association analysis through the study including preparation of transgenic lines and enzyme assay; AF contributed to measurement of cell size and number and made the draft of the manuscript; MM conceived and initiated the project, obtained funding, and contributed to the manuscript. All authors read and approved the final manuscript.

## Conflict of Interest Statement

The authors declare that the research was conducted in the absence of any commercial or financial relationships that could be construed as a potential conflict of interest.

## Abbreviations

DAS: days after sowing
H^+^-PPase: H^+^-translocating pyrophosphatase
PPi: pyrophosphate
VHP: vacuolar H^+^-translocating pyrophosphatase
