# Supplementary material for: Contribution of PPi-Hydrolyzing Function of Vacuolar H+-Pyrophosphatase in Vegetative Growth of Arabidopsis: Evidenced by Expression of Uncoupling Mutated Enzymes
Source: Front Plant Sci. 2016 Mar 31;7:415. doi: 10.3389/fpls.2016.00415 (PMC4814480; doi:10.3389/fpls.2016.00415)
Supplement: Supplementary file 1 [file Data_Sheet_1.PDF]

Supplemental Data

Contribution of PPi-hydrolyzing function of vacuolar H^+-pyrophosphatase in vegetative growth of Arabidopsis: Evidenced by expression of uncoupling mutated enzymes

Mariko Asaoka [1], Shoji Segami [1], Ali Ferjani [2], and Masayoshi Maeshima [1]

[1] Laboratory of Cell Dynamics, Graduate School of Bioagricultural Sciences, Nagoya University, Nagoya 464-8601, Japan, [2] Department of Biology, Tokyo Gakugei University, Nukui Kitamachi 4-1-1, Koganei-shi, Tokyo 184-8501, Japan

Methods

Quantification of Total Triacylglycerol

Experiment was basically carried out according to the method described previously with a few modifications (Ferjani et al. 2011). The total amount of triacylglycerol (TAG) in dry seeds and etiolated seedlings was measured using the assay a Triglyceride E-Test kit (Wako). Either 30 dry seeds or 30 seedlings were homogenized in a mortar in 75 μl of sterile distilled water. Homogenates were mixed with 1,000 μl of reaction buffer of the kit. The concentration of TAG in the sample was determined according to the manufacturer's protocol.

Establishment of Suspension Cultured Cells

Establishment of suspension cultured cells (Mathur and Koncz, 1999) and vacuolar membrane preparation (Segami, et al., 2014) were carried out as described previously with a few modifications. In brief, approximately 1,000 seeds were surface sterilized and placed in 1 ml of water. After agitation for 2 days in the dark at 4°C, we put them into 100 ml of suspension medium (1× MS salt, 2× vitamin B5, 3% sucrose, 0.5 mg/l 2,4-dichlorophenoxyacetic acid, 2 mg/l indole 3-acetic acid, and 0.5 mg/l

6-(γ,γ-dimethylallylamino)-purine riboside, adjust to pH 5.8 with KOH) in a 300 ml flask. The flask was placed on a shaker in the dark at 24 °C. Suspension cultured cells were established after a month. For maintenance of the cell suspension, half of the medium and cells were discarded and fresh suspension medium was added to the remaining culture weekly.

Vacuolar Membrane Preparation from Arabidopsis Cultured Cells

Cells cultured for 150 hours after changing the medium were homogenized at 4°C in a mortar and pestle with sea sand in a 6× volume of homogenizing medium, which contained 0.25 M sorbitol, 50 mM Tris-acetate, pH 7.5, 1 mM EGTA-Tris, 1% polyvinylpyrrolidone, 2 mM dithiothreitol, and protease inhibitor cocktail (1× Complete, EDTA-free, Roche Applied Science). The tissue homogenate was centrifuged at 10,000 × *g* for 10 min at 4°C. The supernatant was centrifuged at 100,000 × *g* for 40 min at 4°C. The pellet was suspended in 33% (w/w) sucrose, 20 mM Tris-acetate, pH 7.5, 1 mM EGTA-Tris, 1 mM $MgCl_2$, and 2 mM dithiothreitol. The suspension was overlaid with 20 mM Tris-acetate, pH 7.5, 0.25 M sorbitol, 1 mM EGTA-Tris, 1 mM $MgCl_2$, and 2 mM dithiothreitol. After centrifugation at 100,000 × *g* for 40 min, the interface portion was collected and diluted with the overlay medium. After centrifugation at 100,000 × *g* for 10 min in a Hitachi S80AT3 rotor, the resulting pellet (tonoplast enriched fraction) was suspended in the overlay medium.

H^+-pumping Assay

PPi-dependent H^+-transport activity was measured as acridine orange fluorescence quenching (Maeshima and Yoshida, 1989) with several alterations. Assay medium (1.9 mL) containing 0.25 M sorbitol, 5 mM MES-Tris (pH 7.2), 50 mM KCl, 0.5 mM dithiothreitol was mixed with 50 µL of sample containing 50 µg VM enriched fraction prepared from suspension cultured cells. Then 1 µM acridine orange and 3 mM $MgCl_2$ were added. The enzyme reaction was initiated by adding 1 mM Na_4PPi. Fluorescence quenching was monitored with a Hitachi F-2700 fluorescent spectrophotometer set at 493 nm for excitation and 540 nm for emission. The reaction was stopped by adding 1.5 mM NH_4Cl.

Reference

Ferjani, A., Segami, S., Horiguchi, G., Muto, Y., Maeshima, M., and Tsukaya, H. (2011). Keep an eye on PPi: the vacuolar-type H^+-pyrophosphatase regulates postgerminative development in *Arabidopsis*. *Plant Cell* 23: 2895–2908. doi: 10.1105/tpc.111.085415

Haydon, M.J., Kawachi, M., Wirtz, M., Hillmer, S., Hell, R. and Krämer, U. (2012). Vacuolar nicotianamine has critical and distinct roles under iron deficiency and for zinc sequestration in Arabidopsis. *Plant Cell* 24: 724–737. doi:10.1105/tpc.111.095042

Maeshima, M., and Yoshida, S. (1989). Purification and properties of vacuolar membrane proton-translocating inorganic pyrophosphatase from mung bean. *J. Biol. Chem.* 264: 20068–20073

Mathur, J., and Koncz, C. (1998). Establishment and Maintenance of Cell Suspension Cultures. Methods in Molecular Biology, Vol. 82: Arabidopsis Protocols, Humana Press, ISBN 0-89603-391-0, p27-30. doi: 10.1385/0-89603-391-0:27

Segami, S., Makino, S., Miyake, A., Asaoka, M., and Maeshima, M. (2014). Dynamics of vacuoles and H^+-pyrophosphatase visualized by monomeric green fluorescent protein in Arabidopsis: artifactual bulbs and native intravacuolar spherical structures. *Plant Cell* 26, 3416–3434. doi: 10.1105/tpc.114.127571

SUPPLEMENTAL TABLE 1 | List of oligonucleotide primer sets

(A) *VHP1* amplification for TOPO cloning

Primer name	DNA sequence (5' - 3')
cacc_VHP1_Fw	caccATGGTGGCGCCTGCTTTGTTACC
VHP1_Rv	TTAGAAGTACTTGAAAAGGATACCACCGTGAGT

(B) Introduction of site-directed mutagenesis

Primer name	DNA sequence (5' - 3')
AtVHP1_I549A_Fw	GTCATTGCTGATAATGTCGG
AtVHP1_I549A_Rv	GCAGCAGAGCCAGCGGCAAATC
AtVHP1_L753A_Fw	GCAGCTATTCCTTTGTGTTTG
AtVHP1_L753A_Rv	GGAGCAAAGACAGCAGACTCAACAGC

Highlighted sequences are sites of substitution to alanine.

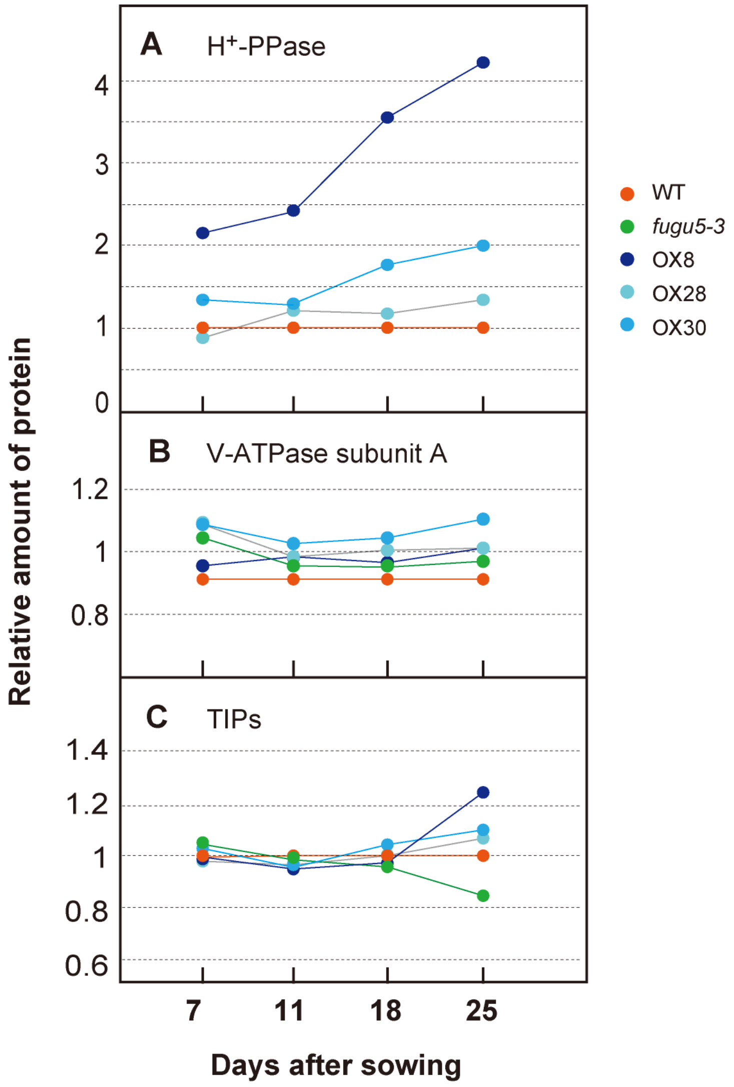

SUPPLEMENTARY FIGURE S1 | Relative protein amount of H^+-PPase, V-ATPase subunit A, and vacuolar membrane aquaporins (TIP1s) in H^+-PPase overexpressors. Crude membrane fractions were prepared from WT, *fugu5-3*, OX8, OX28 and OX30 at indicated growth stages from 7 to 25 DAS. Relative amounts of H^+-PPase (A), V-ATPase subunit A, and TIP1s were determined by immunoblotting using specific antibodies. Data are shown as relative content to WT. DAS, days after sowing.

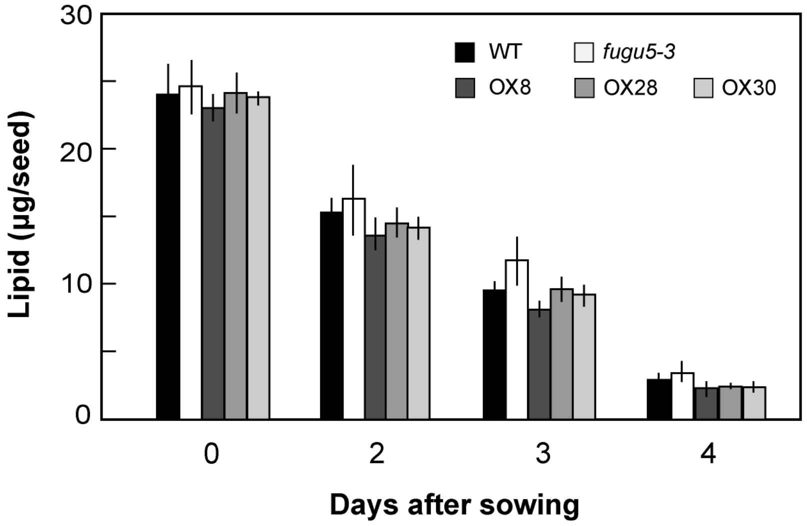

SUPPLEMENTARY FIGURE S2 | Mobilization of seed lipids reserved in H^+-PPase overexpressors. The amounts of triacylglycerol (TAG) were determined. Samples were prepared from dry seeds (day 0) and etiolated seedlings at 2, 3 and 4 DAS. 30 seeds or 30 seedlings were collected per sample set (n = 5). Error bars show SD. DAS, days after sowing.

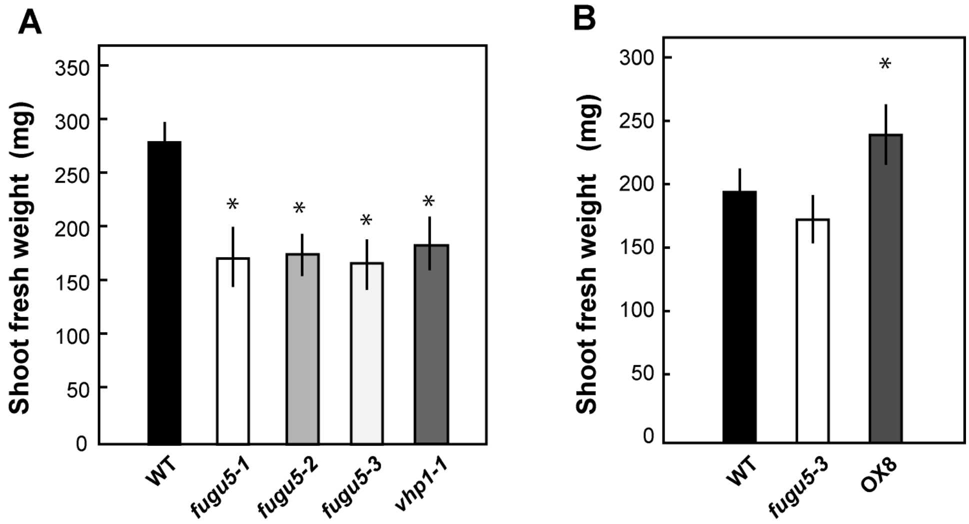

SUPPLEMENTARY FIGURE S3 | Effect of loss-of-function of H^+-PPase. (A) Three *fugu5* mutants (*fugu5-1*, *fugu5-2* and *fugu5-3*) and *vhp1-1* were grown on rockwool pots for 35 days. Shoot weights from WT and mutant lines grown on MS plates were determined. $n = 20$. (B) Shoot fresh weight of 30-day-old plants grown on the MS plates with 1% sucrose. $n = 12$. Error bars show SD. Asterisks indicate significant difference at $P < 0.05$ compared with the WT (Student *t*-test).

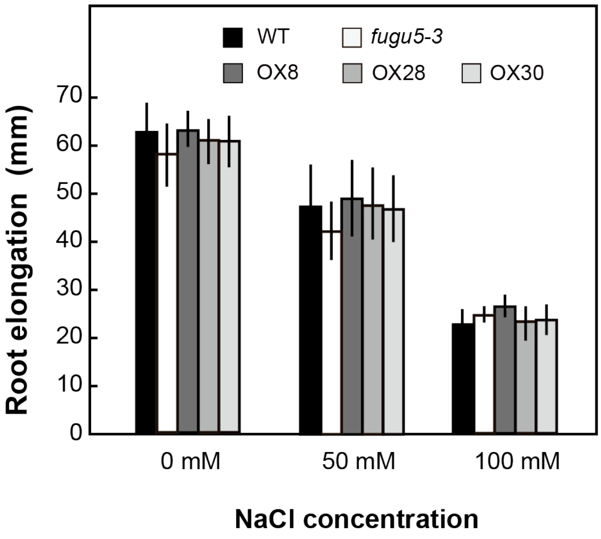

SUPPLEMENTARY FIGURE S4 | Effect of excess NaCl on growth of *fugu5-3* and overexpressors. Seedlings were grown on MS plates with 1% sucrose for 7 days and then transplanted to the plates containing 0 (control), 50 or 100 mM NaCl. Root elongation rate for 6 days in the plates was measured. The values mean the elongation of primary roots for 6 days. $n = 15$.

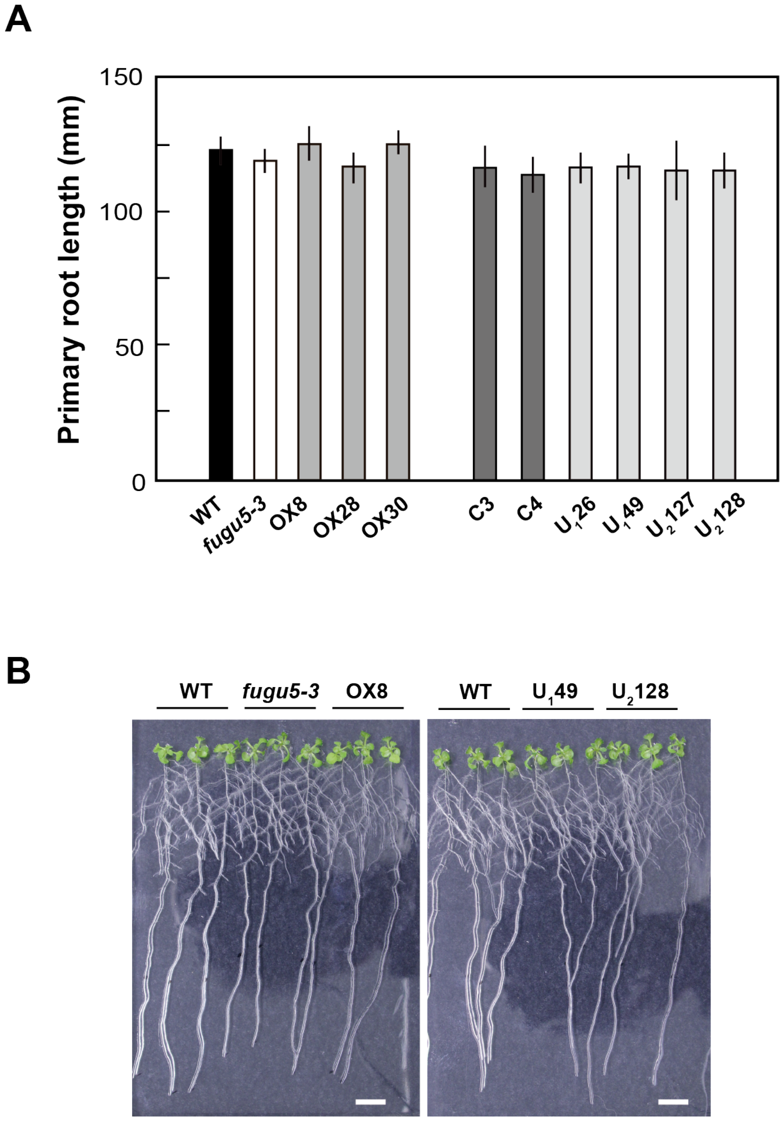

SUPPLEMENTARY FIGURE S5 | Effect of Pi deficiency on growth of *fugu5-3*, overexpressors, and uncoupling mutants. (A) Seedlings were grown in the Hoagland medium (Haydon et al., 2012) for 5 days, transplanted to the Hoagland medium lacking Pi and sucrose, and then grown for another 11 days. Primary root length of 16 day-old plants was measured. *n* = 15. (B) Representative images of 16 day-old plants of WT, *fugu5-3*, OX8, $U_1$49 and $U_2$128, grown under Pi deficient conditions. Scale bar = 10 mm.

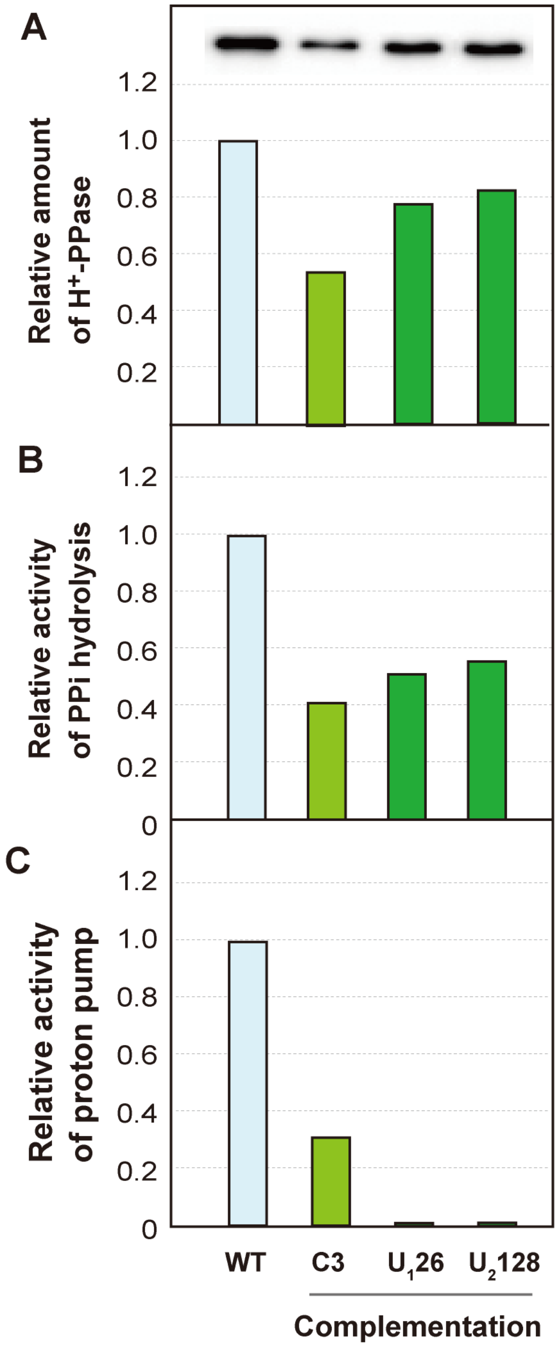

SUPPLEMENTARY FIGURE S6 | Biochemical characteristic of uncoupling H^+-PPase in plant cultured cell system. Cultured cells were established from uncoupling mutated H^+-PPase transgenic plants. Tonoplast enriched fractions were prepared from the suspension cultured cells and subjected to enzymatic assay. Lines C3, $U_1$26 and $U_2$128 were investigated as representative lines. (A) H^+-PPase expression level, (B) PPi hydrolysis activity, (C) H^+-pump activity.
